# Testing Antimicrobial Properties of Human Lactoferrin-Derived Fragments

**DOI:** 10.3390/ijms241310529

**Published:** 2023-06-23

**Authors:** Michał Ostrówka, Anna Duda-Madej, Filip Pietluch, Paweł Mackiewicz, Przemysław Gagat

**Affiliations:** 1 Faculty of Biotechnology, University of Wrocław, Fryderyka Joliot-Curie 14a, 50-137 Wrocław, Poland; michal.ostrowka2@uwr.edu.pl (M.O.); filip.pietluch2@uwr.edu.pl (F.P.); pawel.mackiewicz@uwr.edu.pl (P.M.); 2 Department of Microbiology, Faculty of Medicine, Wrocław Medical University, Chałubińskiego 4, 50-368 Wrocław, Poland; anna.duda-madej@umw.edu.pl

**Keywords:** antimicrobial peptides, bacteria, lactoferrin, non-antimicrobial peptides, prediction, protease inhibitor

## Abstract

Lactoferrin, an iron-binding glycoprotein, plays a significant role in the innate immune system, with antibacterial, antivirial, antifungal, anticancer, antioxidant and immunomodulatory functions reported. It is worth emphasizing that not only the whole protein but also its derived fragments possess antimicrobial peptide (AMP) activity. Using AmpGram, a top-performing AMP classifier, we generated three novel human lactoferrin (hLF) fragments: hLF 397-412, hLF 448-464 and hLF 668-683, predicted with high probability as AMPs. For comparative studies, we included hLF 1-11, previously confirmed to kill some bacteria. With the four peptides, we treated three Gram-negative and three Gram-positive bacterial strains. Our results indicate that none of the three new lactoferrin fragments have antimicrobial properties for the bacteria tested, but hLF 1-11 was lethal against *Pseudomonas aeruginosa*. The addition of serine protease inhibitors with the hLF fragments did not enhance their activity, except for hLF 1-11 against *P. aeruginosa*, which MIC dropped from 128 to 64 µg/mL. Furthermore, we investigated the impact of EDTA with/without serine protease inhibitors and the hLF peptides on selected bacteria. We stress the importance of reporting non-AMP sequences for the development of next-generation AMP prediction models, which suffer from the lack of experimentally validated negative dataset for training and benchmarking.

## 1. Introduction

Lactoferrin (LF) is a member of transferrins, i.e., metal-binding glycoproteins responsible for controlling metal ion levels, especially iron, in biological fluids [1]. This protein with a molecular mass of ~80 kDa was first purified from bovine milk in 1939 [2], and in 1960 it was purified from human milk [3]. The protein consists of two homologous parts designated as N- and C-lobe. The lobes are divided into same-size domains: N1 and N2, and C1 and C2, respectively, and between each pair a ferric ion can be attached [4,5,6]. Presumably, the ancestor of all transferrins, including LF, had only one lobe, but due to a tandem duplication, the double-lobed protein was formed [7,8]. LF is highly glycosylated in a species-dependent manner, which conditions its resistance to low pH, proteolytic degradation and might regulate its other biological properties [9,10,11].

LF has been identified in many mammals, e.g., pigs, buffaloes, horses, sheep, camels, goats, mice and elephants, but the research mostly focuses on bovine (bLF) and human (hLF) proteins [12]. LF is abundant in mammalian saliva, tears, seminal fluid, white blood cells and milk [13,14]. It has been reported to have antibacterial, antiviral, antifungal, anticancer and antioxidant properties but also modulates the immune system [15,16,17].

The antibacterial activity of LF is related to the inhibition of bacterial growth by iron scavenging and its cell membrane disruption. Nutrient metals, including iron, are essential for most pathogenic bacteria as ~30% of their proteins use metal cofactors [18]. Sequestering metals from microorganisms constitutes a key host strategy known as “nutritional immunity”, and LF is part of it [19,20,21]. Additionally, LF due to its highly cationic N-lobe can interact with negatively charged cell wall components of Gram-negative and Gram-positive bacteria, i.e., lipopolysaccharide and lipoteichoic acid, respectively [22]. These bindings trigger the cell wall destabilization and increase the membrane permeability, which can result in the cytoplasmic leakage, disruption of balance of solutes and ions within the cell and ultimately bacterium death [23,24,25].

Antiviral activity of LF has been reported against many viruses, including common diarrhea and cold/flu-causing agents, but also COVID-19, HIV, poliovirus, hepatitis B and C viruses [26,27]. Due to the positive charge, LF may interact with anionic components of eukaryotic cells, including co-receptors for some viruses, e.g., heparan sulfate proteoglycans, or directly bind to virial particles, e.g., the protein spike S in COVID-19. Both interactions prevent virial attachment to the host cells and thereby infection [28,29].

There are also many well-documented cases of antifungal activity of LF; however, the exact mechanism of LF action is not well known [30]. Recent studies suggest that the protein induces both mitochondria- and caspase-dependent regulated apoptosis [31]. Additionally, in both yeast-like fungi and bacteria, LF was shown to bind and inhibit plasma membrane H^+^-ATPase, and consequently to disturb intracellular pH and the transmembrane proton gradient leading to cell death [32].

Apart from its antimicrobial activities, LF has some anticancer and immunomodulatory properties as well [33,34,35]. Its treatment of some cancer cells results in cell membrane disruption, apoptosis, cell cycle arrest and cell immunoreaction, though the exact anticancer mechanisms underlying the LF activity are still vague [34]. By binding to negatively charged regions of various immune cells, LF can regulate their differentiation, maturation and activation, and thereby further regulate the antimicrobial and anticancer response of mammals [28,36].

It is important to emphasize that not only the whole LF protein but also its derived fragments have biological properties (Table 1). Due to their positive charge, hydrophobicity and amphipathicity, they may behave as typical antimicrobial peptides (AMPs), i.e., (i) interact non-specifically with negatively charged components of bacterial cell wall and damage the membrane by solubilization and/or pore formation, and/or (ii) act intracellularly on specific molecules inhibiting proteases, cell division and biosynthesis of nucleic acids [37,38,39,40,41]. Both mechanisms can lead to bacterial cell death and are concentration dependent (Table 1).

The first mechanism has already been indicated for bovine lactoferricin (bLFcin) and lactoferrampin (bLFampin) derived from the bLF N- and C-lobe, respectively (Table 1) [43,54,55]. Peptide bLFcin was also shown to act inside *Escherichia coli* by causing, e.g., pyruvate-associated growth inhibition and bacterial filamentation [48,56]. Additionally, it was revealed that bLFcin binds to response regulators of the two-component system (TCS), BasR and CreB, thereby inhibiting their activation by appropriate cognate kinases [57]. TCS is crucial for protecting the integrity of the bacterial cell wall against antimicrobial molecules, including AMPs [57]. Interestingly, BasR and CreB homologs in probiotic bacteria, e.g., from the genera *Bifidobacterium* and *Lactobacillus*, do not have the amino acid motif targeted by bLFcin. Consequently, this peptide may have marginal influence on microbes beneficial to human health [57].

The human equivalent of bLFcin (hLFcin) corresponds to residues 1 to 47 of hLF. It contains two sub-fragments, hLF 1-11 and hLF 12-47, which are connected by a disulfide bond [49]. Unfortunately, not many studies demonstrate antibacterial properties of the whole hLFcin (Table 1). However, it may have potentially immunomodulatory properties, such as stimulating the release of neutrophil-activating interleukin 8 from leukocytes [58]. More research has been conducted on bLFcin fragments, however. The hLFcin 28–34 loop region was found to exhibit a high affinity for lipopolysaccharide [59] and the hLFcin 17-39 fragment to play a role in depolarizing the *E. coli* cytoplasmic membrane and causing the loss of the pH gradient [60]. Peptide hLF 1-11 is the most extensively studied part of hLFcin with antibacterial activity confirmed against some Gram-positive and Gram-negative bacteria (Table 1). Van Berkel et al. [61] indicated that four consecutive arginine residues of hLF 1-11 play a vital role in its interaction with heparin, lipid A, human lysozyme and DNA, and, furthermore, condition its antimicrobial properties [62]. The peptide was additionally proven to have immunomodulatory properties by stimulating monocytes to produce enhanced levels of pro-inflammatory and anti-inflammatory cytokines and chemokines, probably as a response to the inflammation stage early vs. late, respectively [63].

Taking into account the broad spectrum of activity and the safety of use of LF and its derived peptides, it was approved as an ingredient in food products by the European Food Safety Authority and is commonly added, e.g., to infant formulas [64,65,66,67,68]. We decided to test antimicrobial properties of selected fragments of hLF that have not been investigated yet. To choose suitable candidates for experimental research, we employed AmpGram [69], a top AMP prediction model highly acclaimed in the first unbiased benchmark of AMP predictors [70]. On the basis of AmpGram analyses, we generated three fragments: hLF 397-412, hLF 448-464 and hLF 668-683, and used them to treat six bacterial strains, including three Gram-negative and three Gram-positive species. For comparative studies, we selected hLF 1-11 that has already been confirmed to be antimicrobial for some bacteria (Table 1).

## 2. Results

### 2.1. In silico Selection of Lactoferrin Fragments

**Figure 1:**
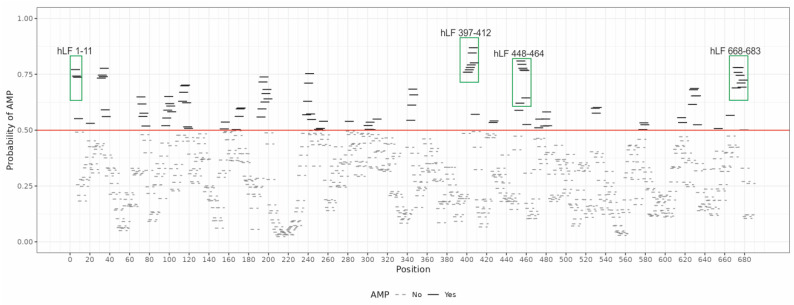
Distribution of AMP or non-AMP 10-mers (sequences of ten amino acids) along the mature human lactoferrin (hLF) sequence (without a signal peptide) produced by AmpGram [69]. The red line indicates the threshold for a 10-mer to be predicted as AMP or non-AMP. The green rectangles mark 10-mers merged to obtain hLF 397-412, hLF 448-464, hLF 668-683 and hLF 1-11 that was used for comparative studies.

We designed three hLF-derived fragments using AmpGram (version 28 February 2022), an AMP prediction model based on n-grams, i.e., short amino acid motifs, and random forests, a machine learning classification method [69]. Like a typical AMP prediction model, AmpGram calculates the probability of a given peptide/protein to be an AMP or a non-AMP. Initially, it divides a peptide/protein into overlapping subsequences of 10 amino acids (10-mers). For each 10-mer, the first random forest model makes a prediction if it is an AMP or a non-AMP. Next, the prediction is scaled for the whole peptide/protein based on statistical analyses of 10-mers with the second random forest model. The second model is responsible for providing the final decision and the probability value. Using the information provided by the first model, AmpGram also draws a chart representing the regions in the amino acid sequence with antimicrobial potential. The chart is generated as AmpGram scans a peptide/protein with the sliding window of ten amino acids in search of n-grams typical of AMPs and non-AMPs. The AMP and non-AMP 10-mers are then plotted along the peptide/protein sequence indicating regions that have an antimicrobial potential [69]. The distribution of 10-mers for hLF is presented in Figure 1. Based on this chart, we selected hLF 397-412, hLF 448-464 and hLF 668-683 by merging the adjacent 10-mers with the highest AMP probability and also hLF 1-11 for comparative studies. The resulting peptides were predicted by AmpGram as AMPs with the probability of 1.0, 0.9817, 0.9862 and 0.9892, respectively (Table 2; for prediction of individual 10-mers see Table S1).

### 2.2. Experimental Verification of Antimicrobial Properties of Lactoferrin Fragments

**Figure 2:**
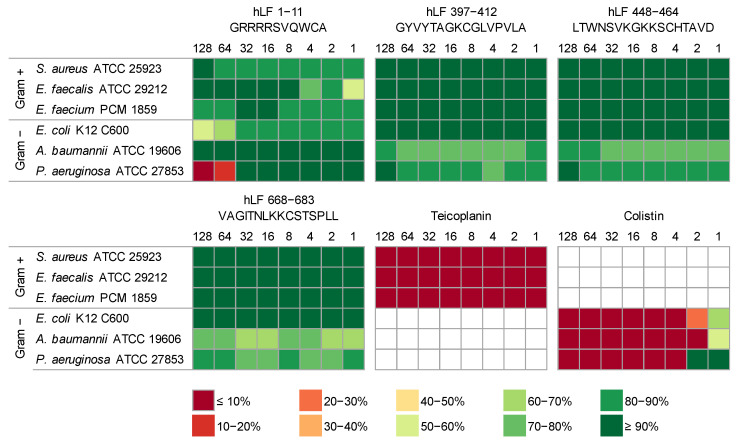
Experimental verification of antimicrobial properties of lactoferrin fragments. Each of the six heat maps shows results for one peptide (hLF) or an antibiotic control (teicoplanin and colistin). The columns represent the decreasing concentration of the tested peptides from 128 to 1 µg/mL, and the rows bacteria used, classified into Gram-negative (Gram−) and Gram-positive (Gram+) groups. The effect of peptides on the bacterial viability is indicated as a color gradient explained under the figure, from dark red (less than 10% bacteria survived) to dark green (more than 90% bacteria survived).

In our research, we evaluated the antimicrobial properties of four hLF-derived peptides, hLF 1-11, hLF 397-412, hLF 448-464 and hLF 668-683, against six reference bacterial strains, three Gram-positive and three Gram-negative. As controls, we used peptide antibiotics colistin [71,72] and teicoplanin [73,74] that were proven to efficiently destroy Gram-negative and Gram-positive bacteria, respectively. Depending on the bacterial strain, the antibiotics displayed MIC values at 4 µg/mL or below (Figure 2).

The results presented in Figure 2 and Tables S2–S7 indicate that among the four investigated peptides, only hLF 1-11 exhibited antimicrobial properties. The MIC value of hLF 1-11 against *P. aeruginosa* was established at 128 µg/mL, which is in agreement with previous reports [75]. This peptide also displayed a moderate bacteriostatic effect against *E. coli*, resulting in its viability at the level of 57%. For these two species, we noticed a significant negative correlation between their viability and the peptide concentration, with the correlation coefficient amounting to −0.77 (*p* = 0.025) and −0.74 (*p* = 0.019), respectively. None of the other tested peptides exhibited antibacterial properties against the studied strains; thus, we were not able to determine their MIC values within the 18 h incubation period for the investigated peptide dilutions (MIC > 128 µg/mL). Nevertheless, *A. baumannii* and *P. aeruginosa* exhibited a slightly smaller viability compared to other bacteria when exposed to hLF 397-412, hLF 448-464 and hLF 668-683 though it was independent of the peptide concentration. We also noticed that the growth of *E. faecalis* was generally very poor, most probably due to the fact that it prefers more enriched media [76].

**Figure 3:**
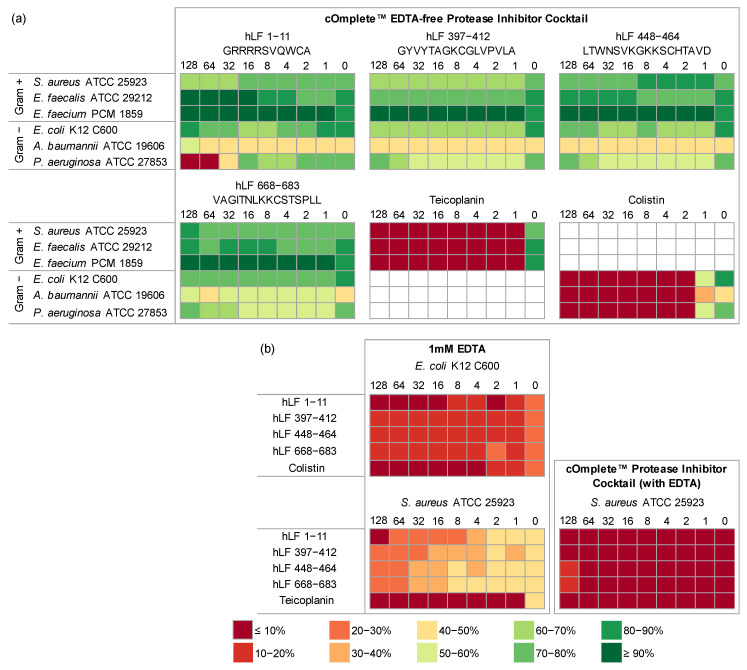
Experimental verification of antimicrobial properties of human lactoferrin (hLF) fragments in combination with cOmplete™ EDTA-free Protease Inhibitor Cocktail at 1× concentration (**a**) and cOmplete™ Protease Inhibitor Cocktail at 1× concentration with EDTA or only 1 mM EDTA (**b**). Each of the six heat maps shows results for one peptide or an antibiotic control. The columns represent peptide dilutions from 128 to 0 µg/mL, and the rows represent bacteria classified into Gram-negative (Gram−) and Gram-positive (Gram+) groups (**a**). Each of the three heat maps shows results for one bacterium. The columns represent peptide dilutions from 128 to 0 µg/mL, and the rows represent peptides or antibiotic control used (**b**). The effect of peptides on the bacterial viability is indicated as a color gradient explained under the figure, from dark red (less than 10% bacteria survived) to dark green (more than 90% bacteria survived).

Moreover, we evaluated the antimicrobial properties of hLF-derived peptides in combination with EDTA-free protease inhibitor cocktail to check if protection from serine proteases would enhance their activity (Figure 3, Tables S8–S13) [77]. Although there was a statistically significant decrease in the viability of most bacteria compared to the corresponding experiments without the inhibitors, it was not very large and did not substantially impact their MIC values, except for hLF 1-11 against *P. aeruginosa*. In this case, the MIC value dropped from 128 µg/mL to 64 µg/mL, with the correlation coefficient between bacterial viability and peptide concentration amounting to −0.86 (*p* = 0.006). What is also worth noting is that the MIC of control antibiotics dropped from 4 µg/mL to 2 µg/mL for *E. coli* and *P. aeruginosa*.

Additionally, we treated *E. coli* and *S. aureus* with 1 mM EDTA to investigate its effect in combination with the studied peptides and antibiotics (Tables S14 and S15). The sole presence of 1 mM EDTA resulted in killing of 80% of *E. coli* culture and 67% of *S. aureus*. The MIC value for hLF 1-11 with EDTA against *E. coli* was 16 µg/mL and that against *S. aureus* 128 µg/mL. For the remaining peptides supplied with EDTA, the MIC values were >128 µg/mL, whereas the bacterial viability ranged from 11 to 20% for *E. coli* and 22 to 47% for *S. aureus*, depending on the peptide concentration. Furthermore, we examined the effect of both hLF-derived peptides and serine protease inhibitors with EDTA on *S. aureus*, and it proved to be lethal independently of the addition of the peptides (Figure 3, Table S16).

## 3. Discussion

In the field of machine learning, particularly in the development of prediction models, the availability of a high-quality training dataset is of paramount importance for obtaining accurate and reliable results [70]. The training dataset usually consists of a positive and a negative subset, and relevant features are extracted from them to feed the machine learning model. In the case of AMP prediction, the positive dataset is composed of AMPs, while the negative one comprises non-AMP sequences. Based on the datasets, models aim to classify whether a given peptide or protein possesses antimicrobial potential or not [70,78]

One of the greatest challenges in developing dependable AMP prediction models is the lack of an experimentally validated negative subset, comprising peptides without any antimicrobial properties; the verified positive subset is easily downloadable from public AMP databases. The vast majority of researchers create their negative dataset by performing non-probability sampling on sequences mostly from UniProt [70]. In this approach, the non-AMPs are selected based on arbitrary defined criteria through sequence filtering, mainly by excluding from the whole set, records with (i) confirmed antimicrobial properties and (ii) secretory peptides as AMPs are generally transported outside the cell. From this pool, sequences are randomly selected for the final negative subset, but some of them could still represent true AMPs as their antimicrobial properties are not verified [70].

The absence of experimentally validated negative data creates several problems. Firstly, without a robust negative dataset, the model may be biased towards positive sequences, resulting in false-positive predictions and reduced accuracy. This means that sequences that are not antimicrobial are indicated as potential AMPs. Secondly, the lack of comprehensive negative data makes it difficult to compare the performance of different models, spreading mistrust of bioinformatic methods. Sidorczuk and Gagat et al. [70] attempted to address the second issue by developing a benchmark methodology for AMP classifiers. However, the first issue can only be resolved by reporting non-AMP sequences. The inclusion of validated non-AMPs in the training dataset is crucial for capturing the diversity and complexity of both AMP and non-AMP peptides and conditions the development of the next-generation AMP prediction models [76].

In our research, we used AmpGram [69,70], a highly effective AMP classifier, and generated three novel human lactoferrin (hLF) fragments, hLF 397-412, hLF 448-464 and hLF 668-683, predicted with high probability to have antimicrobial potential (Figure 1, Table 2). For comparative studies, we employed hLF 1-11 that has already been confirmed to be lethal for some bacteria (Table 1). Interestingly, none of the three new lactoferrin fragments exhibited any antimicrobial properties against the six tested bacteria, including three Gram-negative and three Gram-positive species (Figure 2). Considering the possibility that bacterial proteases could be responsible for degrading the hLF fragments, we further tested their antimicrobial potential using a EDTA-free protease inhibitor cocktail, containing 4-(2-aminoethyl)benzenesulfonyl fluoride hydrochloride (AEBSF). AEBSF is a member of sulfonyl fluorides, which selectively and irreversibly inhibit serine proteases by reacting with the hydroxyl group of serine residues in the enzyme active site. Consequently, it might provide the investigated peptides with some protection against proteolysis [77]. However, the application of AEBSF did not substantially decrease the viability of the tested bacteria subjected to the lactoferrin-derived peptides, which means that hLF 397-412, hLF 448-464 and hLF 668-683 do not display significant antimicrobial properties against them. Their weaker antimicrobial activity compared to hLF 1-11 might be associated with smaller content or segment of positively charged residues; hLF 1-11 consists of four consecutive arginine residues, whereas the new hLF peptides have up to two lysine residues.

We also investigated the effect of the hLF-derived peptides with 1 mM EDTA. Similarly to lactoferrin, EDTA has been reported to sequester metal ions from microorganisms, including calcium and magnesium, which are essential for functioning of bacterial enzymes, e.g., proteases, and maintaining the stability and integrity of the bacterial cell wall, especially of Gram-negative bacteria [78,79]. Accordingly, the sole presence of 1 mM EDTA resulted in killing most of *E. coli* culture and a substantial part of *S. aureus* (Figure 3). However, the addition of the hLF fragments in these experiments only slightly decreased the bacterial viability. Interestingly, the combination of AEBSF and 1 mM EDTA triggered the complete destruction of the *S. aureus* culture (Figure 3), most probably due to the synergistic effect of both compounds [80]

The only hLF-derived fragment in our studies that displayed antimicrobial potential was hLF 1-11. It proved to be lethal for *P. aeruginosa* at 128 µg/mL and moderately bacteriostatic against *E. coli*. Its antimicrobial effect was further strengthened in combination with EDTA-free protease inhibitor cocktail (MIC 64 µg/mL) and EDTA (MIC 16 µg/mL) against *E. coli*. Given their mechanisms of action, AEBSF could provide hLF 1-11 with some protection from proteolysis, whereas EDTA not only protects this peptide but also increases the bacterial wall permeability [79,80,81].

Our results provide valuable insights into the antimicrobial activity of hLF-derived peptides and highlight the problem of false-positive predictions and reduced accuracy among AMP prediction models, including AmpGram [69]. The models fall short of their full potential, but they can be easily improved by inclusion of experimentally validated non-AMP sequences into their negative datasets used for model training. We are aware that the impact of our study is limited because we provide only three non-AMPs though hundreds are necessary to make a real difference in the field. However, the three peptides we verified in vitro were derived from an extensively studied protein—hLF, and therefore they are important to have been investigated. Furthermore, we would like to further encourage researchers to join our effort and add their own non-AMPs to the growing list in Table S17.

## 4. Materials and Methods

### 4.1. Sequences

The computationally selected peptides for experimental research, hLF 397-412 (GYVYTAGKCGLVPVLA), hLF 448-464 (LTWNSVKGKKSCHTAVD) and hLF 668-683 (VAGITNLKKCSTSPLL), were synthetized by Lipopharm.pl (Gdańsk, Poland), and hLF 1-11 (GRRRRSVQWCA) was synthetized by CASLO ApS (Kongens Lyngby, Denmark). All peptides were synthetized with purities greater than 95%.

### 4.2. Strains and Culture Conditions

Six non-pathogenic bacterial strains, three Gram-positive—*Staphylococcus aureus* ATCC 25923, *Enterococcus faecalis* ATCC 29212, *Enterococcus faecium* PCM 1859—and three Gram-negative—*Acinetobacter baumannii* ATCC 19606, *Escherichia coli* K12 C600*, Pseudomonas aeruginosa* ATCC 27853—were used in the study. All strains were obtained from the American Type Culture Collection (ATTC), but *E. faecium* came from the Polish Collection of Microorganisms (PCM).

All bacterial strains were stored at −80 °C. The strains were revived using overnight cultures prepared in the Tryptic Soy Broth (TSB, OXOID, Basingstoke, United Kingdom) and incubated under shaking conditions (MaxQTM6000 incubator shaker, Thermo Scientific, Waltham, MA, USA) at 125 rpm and 37 °C. After that, the purity of strains was checked using enriched media appropriate for the tested strains. For each experiment, a fresh 18–20 h culture was prepared on the Tryptic Soy Agar (TSA, OXOID, Basingstoke, United Kingdom) for Gram-positive bacteria and MacConkey Agar (MC, OXOID, Basingstoke, United Kingdom) for Gram-negative bacteria. Bacteria were then transferred to fresh Muller Hinton Broth (MHB, OXOID, Basingstoke, United Kingdom), where their density was determined. Each culture was established at 10^8^ CFU/mL and then diluted to a starting inoculum of 10^6^ CFU/mL.

### 4.3. Determination of the Antimicrobial Properties of Lactoferrin Fragments

The microdilution method was used to measure the antimicrobial activity of hLF-derived peptides. The test was performed according to the recommendations of the European Antimicrobial Susceptibility Testing Committee [82].

In 384-well microtiter plates, dilutions of the tested hLF fragments were prepared from 128 to 1 µg/mL in the MHB medium from the 1 mg/mL hLF stock solutions in MHB. The bacterial suspension prepared in MHB with a density of 10^6^ CFU/mL was added to 30 µL of MHB containing the appropriate concentrations of AMP, thus obtaining a final bacterial concentration of 10^5^ CFU/mL in the total volume of 60 µL. Pure MHB was used as the blank control, while the control for bacterial growth included an appropriate bacterial strain grown in the MHB medium. For the positive controls, we employed the following antibiotics: colistin [71] for Gram-negative bacteria and teicoplanin [73] for Gram-positive bacteria instead of LF-derived peptides. Microtiter plates were incubated at 37 °C for 18 h. Absorbance (Abs_600nm_) was measured in a microplate reader (Multiskan FC Microplate Photometer; Thermo Scientific, Waltham, MA, USA). The absorbance results were normalized to the percentages with respect to the growth control. The MIC for each peptide was defined as the concentration that kills more than 90% of the initial inoculum within 18 h. We performed three independent studies for all bacterial strains, each study representing a different bacterial colony, and each colony was tested in three independent repetitions.

Furthermore, we investigated the combined impact of LF-derived fragments with protease inhibitors on bacteria to check if their protection would enhance peptide activity. We used cOmplete™ EDTA-free Protease Inhibitor Cocktail (Roche, Basel, Switzerland) at a 1× concentration in each well in a total volume of 60 µL. All bacteria and peptide solutions were prepared as described above, but we performed two independent studies for all bacterial strains in three independent repetitions. We also studied the effect of LF-derived fragments together with (i) EDTA at the final concentration of 1 mM on *E. coli* and *S. aureus*, and (ii) cOmplete™ Protease Inhibitor Cocktail by Roche at a 1× concentration containing 1 mM EDTA on *S. aureus*. These experiments were performed only once in three independent repetitions each.

Pearson correlation coefficient was calculated between bacteria viability and the logarithm of peptide concentration. Paired Student’s *t*-test and Wilcoxon test (depending on fulfilling the normal distribution by the data) were applied to verify the difference between viability of bacteria subjected to peptides without and with protease inhibitors. The analyses were conducted in STATISTICA (TIBCO_Software_Inc., Palo Alto, CA, USA, 2017, version 13).

## 5. Conclusions

LF and its derived peptides have been intensively investigated for their diverse biological activities, including antibacterial, antiviral, antifungal, anticancer and immunomodulatory potential. They display their antimicrobial properties through iron scavenging, cell membrane disruption, and interaction with microorganism intracellular components. In our research, we verified the antimicrobial effect of previously unexplored fragments of hLF, along with hLF 1-11 tested for comparative studies; hLF 1-11 has already been confirmed to kill some bacteria. We generated three hLF-derived peptides, hLF 397-412, hLF 448-464 and hLF 668-683, using a reliable AMP prediction model AmpGram. Our results indicate that only hLF 1-11 demonstrated significant antibacterial activity (MIC ≤ 128 µg/mL). The addition of serine protease inhibitors did not substantially enhance the antibacterial properties of hLF fragments, except for hLF 1-11 against *P. aeruginosa*, which means that the proteolytic degradation does not account for their lack of antimicrobial qualities. Furthermore, we showed that the presence of EDTA has a detrimental effect on bacterial viability, but the antimicrobial effect was only slightly strengthened in combination with the investigated peptides. Our findings contribute to a deeper understanding of LF and hLF-derived peptides, and provide three non-AMPs for the development of the next-generation AMP prediction models.

## Figures and Tables

**Table 1:** Selected bovine (bLF) and human (hLF) lactoferrin-derived fragments with antibacterial properties. MIC—minimum inhibitory concentration for each peptide, given in round brackets. *A. baumannii—Acinetobacter baumannii*; *B. cereus*—*Bacillus cereus*; *B. subtilis*—*Bacillus subtilis*; *C. perfringens*—*Clostridium perfringens*; *C.renale—Corynebacterium renale; E. faecalis*—*Enterococcus faecalis*; *E. coli*—*Escherichia coli*; *K. pneumoniae*—*Klebsiella pneumoniae*; *L. monocytogenes*—*Listeria monocytogenes*; *P. intermedia—Prevotella intermedia*; *P. vulgaris*—*Proteus vulgaris*; *P. aeruginosa*—*Pseudomonas aeruginosa*; *S. aureus*—*Staphylococcus aureus*; *S. mutans*—*Streptococcus mutans*; *S. salivarius—Streptococcus salivarius*; *S. sobrinus*—*Streptococcus sobrinus*; *S.* Typhimurium—Salmonella enterica subsp. enterica serovar Typhimurium; *S.* Montevideo—Salmonella enterica subsp. enterica serovar Montevideo; *S.* Salford—Salmonella enterica subsp. enterica serovar Salford; *S.* Enteritidis—Salmonella enterica subsp. enterica serovar Enteritidis; *Y. enterocolitica—Yersinia enterocolitica*.

Peptide	Gram-positive (+) and Gram-negative (−) Bacterial Strain (MIC-µg/mL)		Ref
bLF 1-20	*+B. subtilis* NBRC 3134 (50)	*−E. coli* NBRC 3301 (50)	[42]
bLF 17-30	*+S. aureus* ATCC 25923 (76.8) *+* MRSA (38.4)	−*E. coli* EPEC E2348/69 (76.8) −*E. coli* MREPEC (38.4) −*E. coli* EHEC O157:H7 (38.4)	[43]
bLF 17-30	*+S. aureus* HG386 (75) *+S. mutans* HG455 (37) *+S. sobrinus* OB50 (37) *+S. salivarius* HG475 (37)	*−E. coli* K12 (37) *−K. pneumoniae* HG389 (75) *−P. intermedia* OB51 (18)	[44]
bLF 20-30	*+B. subtilis* NBRC 3134 (6.25)	*−E. coli* NBRC 3301 (25)	[42]
bLF 17-31	*+E. faecalis* ATCC 29212 (50)	*−E. coli* ATCC 25922 (50)	[45]
bLF 19-37		*−P. intermedia* OB51 (75)	[44]
bLF 17-41 (bLFcin)	*+L. monocytogenes* EGD (1.6) *+L. monocytogenes* 4b maritime (6.6) *+S. aureus* ATCC 29213 (6.6)	*−E. coli* K-12 (1.6) ^1^ *−E. coli* CL99 (8.5) ^1^ *−S*. Typhimurium SL696 (13) ^1^ *−S.* Montevideo SL5222 (8) ^1^ *−E. coli* ATCC 25922 (3.3) *−P. aeruginosa* ATCC 2783 (3.3) *−P. aeruginosa* PAO-1 (3.3)	[46]
	*+B. cereus* ACM 446 (18.75) *+S. aureus* NCTC 6571 (18.75) *+L. monocytogenes* NCTC 7973 (6.25)	*−E. coli* O:9 (18.75) *−E. coli* L361 (12.5) *−S.* Salford IMVS 1710 (12.5)	[47]
		*−E. coli* ATCC 25922 (30)	[48]
	*+E. faecalis* ATCC 29212 (100)	*−E. coli* ATCC 25922 (100)	[45]
	*+S. aureus* JCM-2179 (3) *+S. mutans* JCM-5705T (2) *+L. monocytogenes* JCM-7673 (0.3) *+C. renale* JCM-1322 (0.6) *+B. subtilis* ATCC-6633 (0.6) *+C. perfringens* ATCC-6013 (12)	*−E. coli* 0111 (6) *−E. coli* IIO-861 (6) *−S.* Enteritidis 110-604 (12) *−K. pneumoniae* JCM-i662T (9) *−P. vulgaris* JCM-i668T (12) *−Y. enterocolitica* 110-981 (6) *−P. aeruginosa* IFO-3446 (9)	[49]
bLF 265-284 (bLFampin)	*+S. aureus* ATCC 25923 (47.8) + MRSA (47.8)	*−E. coli* EPEC E2348/69 (47.8) *−E. coli* MREPEC (47.8) *−E. coli* EHEC O157:H7 (95.6)	[43]
bLF 269-288	*+B. subtilis* NBRC 3134 (50)	*−E. coli* NBRC 3301 (50)	[42]
bLF 343-351	*+B. subtilis* NBRC 3134 (50)	*−E. coli* NBRC 3301 (100)	[42]
bLF chimera ^2^	*+S. aureus* ATCC25923 (15.125) *+E. faecalis* ATCC 29212 (15.125) *+L. monocytogenes* ATCC 19111 (7.56)	*−E. coli* ATCC 25922 (30.25) *−S.* Typhimurium ATCC 14028 (7.56)	[50]
	*+S. aureus* ATCC 25923 (4.4) + MRSA (43.8)	*−E. coli* EPEC E2348/69 (4.4) *−E. coli* MREPEC (4.4) *−E. coli* EHEC O157:H7 (4.4)	[43]
hLF 1-11	*+S. aureus* 2141 (6.85) *+L. monocytogenes* EGD (6.85)	*−K. pneumoniae* ATCC 43816 (6.85) *−E. coli* O54 (6.85)	[51]
		−*A. baumannii* LUH 6034, 7858, 7312 (12.33) −*A. baumannii* RUH 875, 134 (12.33)	[52]
	*+E. faecalis* ATCC 29212 (100)	*−E. coli* ATCC 25922 (25)	[45]
		−*A. baumannii* (40)	[53]
hLF 17-30		*−P. intermedia* OB51 (18)	[44]
hLF 1-47 (hLFcin)		*−E. coli* 0111 (100)	[49]
hLF hydrolysate		*−E. coli* 0111 (100)	[49]

^1^ mean value from different preparations; ^2^ peptide derived from combining bLF 17-30 and bLF 265-284 fragments.

**Table 2:** Human lactoferrin (hLF) fragments selected for in vitro studies based on AmpGram prediction (AMP probability). MW—molecular weight, pI—isoelectric point.

Peptide	Sequence	MW	pI	AMP Probability
hLF 1-11	GRRRRSVQWCA	1374.59	12.00	0.9892
hLF 397-412	GYVYTAGKCGLVPVLA	1610.93	8.18	1.0000
hLF 448-412	LTWNSVKGKKSCHTAVD	1874.14	9.20	0.9817
hLF 668-683	VAGITNLKKCSTSPLL	1644.99	9.31	0.9862

## Data Availability

The data presented in this study are available in supplementary materials.

## Supplementary Materials

The following supporting information can be downloaded at: https://www.mdpi.com/article/10.3390/ijms241310529/s1.
