# Supplementary material for: Testing Antimicrobial Properties of Human Lactoferrin-Derived Fragments
Source: Int J Mol Sci. 2023 Jun 23;24(13):10529. doi: 10.3390/ijms241310529 (PMC10342102; doi:10.3390/ijms241310529)
Supplement: Supplementary file 1 [file ijms-24-10529-s001.zip › ijms-2448014-supplementary.pdf]

Article

Supplementary Materials

Testing Antimicrobial Properties of Human Lactoferrin Fragments

Michał Ostrówka [1], Filip Pietluch [1], Anna Duda-Madej [2], Paweł Mackiewicz [1] and Przemysław Gagat [1]*

[1] Faculty of Biotechnology, University of Wrocław, Fryderyka Joliot-Curie 14a, 50-137 Wrocław, Poland; michal.ostrowka2@uwr.edu.pl; filip.pietluch2@uwr.edu.pl; pawel.mackiewicz@uwr.edu.pl; przemyslaw.gagat@uwr.edu.pl

[2] Department of Microbiology, Faculty of Medicine, Wrocław Medical University, Chałubińskiego 4, 50-368 Wrocław, Poland; anna.duda-madej@umw.edu.pl

* Correspondence: przemyslaw.gagat@uwr.edu.pl

Table S1. Prediction of invidual 10-mers used to design hLF 397-412, hLF 448-412 and hLF 668-683. The 10-mer number represents the order in which each 10-mer was predicted, from the highest 1 to the lowest 114.

hLF fragment	10-mer sequence	AMP probability	10-mer number
hLF 397-412	AGKCGLVPVL	0.8690	1
	TAGKCGLVPV	0.8455	2
	GKCGLVPVLA	0.8013	4
	YTAGKCGLVP	0.7921	6
	VYTAGKCGLV	0.7811	7
	YVYTAGKCGL	0.7701	13
	GYVYTAGKCG	0.7596	16
hLF 448-412	WNSVKGKKSC	0.8097	3
	NSVKGKKSCH	0.7947	5
	SVKGKKSCHT	0.7769	10
	VKGKKSCHTA	0.7692	14
	KGKKSCHTAV	0.7672	15
	GKKSCHTAVD	0.6445	46
	TWNSVKGKKS	0.6207	52
	LTWNSVKGKK	0.5890	65
hLF 668-683	VAGITNLKKC	0.7810	8
	GITNLKKCST	0.7805	9
	AGITNLKKCS	0.7584	18
	ITNLKKCSTS	0.7453	21
	LKKCSTSPLL	0.7239	27
	TNLKKCSTSP	0.7111	29
	NLKKCSTSPL	0.6925	33
	YVAGITNLKK	0.6890	34

Table S2. Absorbance values for *Staphylococcus aureus* ATCC 25923 measured in the MHB medium with the addition of a given human lactoferrin (hLF)-derived peptide or teicoplanin control antibiotic. Numbers from 128 to 1 represent peptide dilutions in µg/mL. Each colony has its own growth control. Growth control 1 corresponds to hLF 1-11 while Growth control 2 to hLF 397-412, hLF 448-464, hLF 668-683 and teicoplanin. R1; R2; R3 – indicate repetitions. C – relates to the mean growth control for each colony.

S. aureus		**Colony 1**								**Colony 2**								**Colony 3**							
		**128**	**64**	**32**	**16**	**8**	**4**	**2**	**1**	**128**	**64**	**32**	**16**	**8**	**4**	**2**	**1**	**128**	**64**	**32**	**16**	**8**	**4**	**2**	**1**
**hLF 1-11**	**R1**	0.64	0.63	0.56	0.62	0.65	0.53	0.63	0.61	0.64	0.65	0.57	0.49	0.51	0.60	0.60	0.55	0.62	0.55	0.56	0.58	0.49	0.61	0.67	0.62
	**R2**	0.67	0.63	0.51	0.58	0.48	0.48	0.54	0.56	0.54	0.55	0.47	0.48	0.53	0.48	0.54	0.52	0.57	0.53	0.56	0.64	0.55	0.51	0.53	0.67
	**R3**	0.58	0.55	0.45	0.55	0.48	0.54	0.49	0.50	0.55	0.52	0.49	0.49	0.48	0.53	0.51	0.50	0.54	0.52	0.49	0.58	0.49	0.54	0.59	0.55
**Growth control 1**	**C**	0.59±0.03								0.66±0.04								0.68±0.02							
**hLF 397-412**	**R1**	0.54	0.54	0.49	0.48	0.51	0.49	0.48	0.42	0.48	0.56	0.46	0.48	0.46	0.46	0.45	0.50	0.47	0.48	0.51	0.43	0.47	0.49	0.57	0.52
	**R2**	0.56	0.54	0.49	0.49	0.48	0.59	0.45	0.46	0.52	0.49	0.46	0.45	0.47	0.42	0.41	0.45	0.45	0.48	0.46	0.48	0.54	0.50	0.55	0.54
	**R3**	0.52	0.53	0.50	0.53	0.50	0.47	0.48	0.47	0.49	0.54	0.49	0.52	0.48	0.48	0.46	0.47	0.52	0.47	0.48	0.48	0.46	0.50	0.52	0.53
**hLF 448-464**	**R1**	0.62	0.62	0.61	0.55	0.55	0.54	0.50	0.52	0.63	0.64	0.61	0.57	0.56	0.56	0.53	0.52	0.61	0.59	0.61	0.60	0.58	0.57	0.57	0.55
	**R2**	0.61	0.53	0.57	0.60	0.56	0.55	0.53	0.52	0.59	0.62	0.61	0.60	0.58	0.57	0.54	0.51	0.58	0.60	0.61	0.57	0.60	0.58	0.57	0.55
	**R3**	0.66	0.69	0.68	0.59	0.57	0.56	0.55	0.53	0.66	0.65	0.62	0.59	0.57	0.54	0.51	0.52	0.61	0.64	0.61	0.57	0.57	0.59	0.59	0.66
**hLF 668-683**	**R1**	0.56	0.57	0.50	0.47	0.46	0.47	0.53	0.51	0.53	0.53	0.56	0.53	0.51	0.50	0.50	0.50	0.54	0.53	0.53	0.50	0.52	0.50	0.54	0.55
	**R2**	0.56	0.52	0.50	0.48	0.48	0.47	0.46	0.45	0.52	0.54	0.50	0.51	0.48	0.50	0.53	0.50	0.52	0.54	0.51	0.53	0.52	0.56	0.56	0.58
	**R3**	0.56	0.56	0.51	0.48	0.50	0.52	0.49	0.50	0.61	0.52	0.51	0.51	0.49	0.52	0.53	0.51	0.50	0.53	0.52	0.51	0.53	0.52	0.54	0.53
**Teicoplanin**	**R1**	0.00	0.00	0.00	0.00	0.00	0.00	0.00	0.00	0.00	0.00	0.00	0.00	0.00	0.00	0.00	0.00	0.00	0.00	0.00	0.00	0.00	0.00	0.00	0.00
	**R2**	0.00	0.00	0.00	0.00	0.00	0.00	0.00	0.00	0.00	0.00	0.00	0.00	0.00	0.00	0.00	0.00	0.00	0.00	0.00	0.00	0.00	0.01	0.00	0.00
	**R3**	0.00	0.00	0.00	0.00	0.00	0.00	0.00	0.00	0.00	0.00	0.00	0.00	0.00	0.00	0.00	0.00	0.00	0.00	0.00	0.00	0.00	0.00	0.00	0.00
**Growth control 2**	**C**	0.55±0.02								0.50±0.04								0.55±0.01							

Table S3. Absorbance values for *Enterococcus faecalis* ATCC 29212 measured in the MHB medium with the addition of a given human lactoferrin (hLF)-derived peptide or teicoplanin control antibiotic. Numbers from 128 to 1 represent peptide dilutions in µg/mL. Each colony has its own growth control. Growth control 1 corresponds to hLF 1-11 while Growth control 2 to hLF 397-412, hLF 448-464, hLF 668-683 and teicoplanin. R1; R2; R3 – indicate repetitions. C – relates to the mean growth control for each colony.

E. faecalis		**Colony 1**								**Colony 2**								**Colony 3**							
		**128**	**64**	**32**	**16**	**8**	**4**	**2**	**1**	**128**	**64**	**32**	**16**	**8**	**4**	**2**	**1**	**128**	**64**	**32**	**16**	**8**	**4**	**2**	**1**
**hLF 1-11**	**R1**	0.25	0.20	0.20	0.19	0.11	0.10	0.08	0.06	0.16	0.19	0.18	0.13	0.10	0.08	0.06	0.06	0.19	0.13	0.19	0.17	0.10	0.08	0.08	0.10
	**R2**	0.25	0.23	0.18	0.18	0.10	0.08	0.09	0.07	0.18	0.21	0.21	0.19	0.17	0.06	0.10	0.04	0.17	0.19	0.18	0.12	0.19	0.10	0.16	0.07
	**R3**	0.26	0.23	0.20	0.16	0.13	0.09	0.09	0.07	0.20	0.25	0.23	0.13	0.16	0.12	0.12	0.06	0.20	0.16	0.21	0.20	0.16	0.19	0.14	0.13
**Growth control 1**	**C**	0.12±0.03								0.13±0.01								0.13±0.01							
**hLF 397-412**	**R1**	0.17	0.17	0.17	0.17	0.16	0.14	0.14	0.13	0.17	0.18	0.16	0.16	0.14	0.14	0.12	0.14	0.18	0.16	0.13	0.16	0.12	0.07	0.06	0.08
	**R2**	0.18	0.13	0.18	0.15	0.15	0.13	0.12	0.13	0.19	0.19	0.19	0.17	0.15	0.13	0.11	0.11	0.19	0.18	0.18	0.11	0.19	0.11	0.16	0.12
	**R3**	0.18	0.17	0.19	0.16	0.14	0.15	0.16	0.14	0.19	0.18	0.18	0.16	0.16	0.14	0.14	0.15	0.19	0.19	0.17	0.16	0.12	0.08	0.09	0.05
**hLF 448-464**	**R1**	0.23	0.22	0.22	0.21	0.17	0.18	0.18	0.17	0.22	0.22	0.18	0.19	0.17	0.14	0.16	0.14	0.21	0.21	0.19	0.20	0.16	0.12	0.11	0.04
	**R2**	0.18	0.18	0.21	0.16	0.16	0.18	0.10	0.13	0.17	0.21	0.20	0.15	0.17	0.15	0.13	0.09	0.18	0.08	0.18	0.14	0.15	0.10	0.12	0.10
	**R3**	0.24	0.23	0.22	0.20	0.18	0.17	0.16	0.16	0.18	0.20	0.20	0.14	0.13	0.11	0.11	0.14	0.11	0.16	0.15	0.14	0.12	0.12	0.11	0.04
**hLF 668-683**	**R1**	0.18	0.11	0.17	0.12	0.17	0.12	0.16	0.09	0.16	0.14	0.19	0.17	0.14	0.16	0.11	0.15	0.19	0.20	0.19	0.17	0.17	0.15	0.16	0.10
	**R2**	0.14	0.14	0.12	0.15	0.15	0.11	0.17	0.16	0.20	0.22	0.17	0.15	0.17	0.16	0.15	0.13	0.18	0.20	0.18	0.18	0.14	0.12	0.08	0.05
	**R3**	0.19	0.18	0.20	0.18	0.18	0.18	0.16	0.11	0.19	0.18	0.17	0.17	0.17	0.14	0.10	0.12	0.20	0.20	0.19	0.13	0.15	0.10	0.16	0.11
**Teicoplanin**	**R1**	0.00	0.00	0.00	0.00	0.00	0.00	0.00	0.00	0.00	0.00	0.00	0.00	0.00	0.00	0.00	0.00	0.00	0.00	0.00	0.00	0.00	0.00	0.00	0.00
	**R2**	0.00	0.00	0.00	0.00	0.00	0.00	0.00	0.00	0.00	0.00	0.00	0.00	0.00	0.00	0.00	0.00	0.00	0.00	0.00	0.00	0.00	0.00	0.00	0.00
	**R3**	0.00	0.00	0.00	0.00	0.00	0.00	0.00	0.00	0.00	0.00	0.00	0.00	0.00	0.00	0.00	0.00	0.00	0.00	0.00	0.00	0.00	0.00	0.00	0.00
**Growth control 2**	**C**	0.12±0.01								0.10±0.01								0.12±0.01							

Table S4. Absorbance values for *Enterococcus faecium* PCM1859 measured in the MHB medium with the addition of a given human lactoferrin (hLF)-derived peptide or teicoplanin control antibiotic. Numbers from 128 to 1 represent peptide dilutions in µg/mL. Each colony has its own growth control. Growth control 1 corresponds to hLF 1-11 while Growth control 2 to hLF 397-412, hLF 448-464, hLF 668-683 and teicoplanin. R1; R2; R3 – indicate repetitions. C – relates to the mean growth control for each colony.

E. faecium		**Colony 1**								**Colony 2**								**Colony 3**							
		**128**	**64**	**32**	**16**	**8**	**4**	**2**	**1**	**128**	**64**	**32**	**16**	**8**	**4**	**2**	**1**	**128**	**64**	**32**	**16**	**8**	**4**	**2**	**1**
**hLF 1-11**	**R1**	0.43	0.41	0.47	0.43	0.40	0.40	0.36	0.39	0.39	0.39	0.39	0.36	0.35	0.33	0.34	0.35	0.39	0.40	0.38	0.36	0.37	0.36	0.37	0.40
	**R2**	0.41	0.40	0.44	0.40	0.40	0.38	0.43	0.39	0.37	0.36	0.39	0.38	0.35	0.34	0.39	0.37	0.38	0.38	0.38	0.37	0.38	0.39	0.41	0.41
	**R3**	0.41	0.42	0.49	0.46	0.44	0.39	0.40	0.41	0.38	0.37	0.40	0.40	0.39	0.39	0.37	0.40	0.38	0.37	0.40	0.41	0.40	0.38	0.40	0.41
**Growth control 1**	**C**	0.44±0.04								0.42±0.01								0.46±0.01							
**hLF 397-412**	**R1**	0.44	0.41	0.38	0.33	0.31	0.26	0.26	0.22	0.38	0.37	0.27	0.28	0.27	0.24	0.25	0.24	0.38	0.33	0.33	0.32	0.25	0.26	0.19	0.23
	**R2**	0.45	0.41	0.39	0.33	0.28	0.27	0.26	0.21	0.40	0.37	0.29	0.32	0.33	0.28	0.24	0.24	0.33	0.27	0.29	0.29	0.26	0.25	0.24	0.26
	**R3**	0.44	0.37	0.39	0.32	0.30	0.27	0.24	0.23	0.35	0.35	0.28	0.32	0.28	0.24	0.27	0.25	0.43	0.35	0.27	0.30	0.31	0.29	0.22	0.21
**hLF 448-464**	**R1**	0.44	0.42	0.37	0.38	0.32	0.29	0.31	0.30	0.44	0.40	0.36	0.33	0.30	0.28	0.28	0.30	0.45	0.42	0.41	0.37	0.28	0.31	0.33	0.32
	**R2**	0.46	0.40	0.41	0.32	0.29	0.31	0.30	0.30	0.44	0.38	0.32	0.32	0.31	0.32	0.27	0.33	0.49	0.42	0.36	0.34	0.34	0.31	0.28	0.31
	**R3**	0.48	0.45	0.46	0.41	0.35	0.30	0.31	0.30	0.47	0.40	0.40	0.32	0.33	0.28	0.31	0.31	0.47	0.41	0.40	0.39	0.33	0.29	0.31	0.31
**hLF 668-683**	**R1**	0.53	0.37	0.40	0.34	0.36	0.30	0.32	0.26	0.43	0.33	0.33	0.31	0.31	0.31	0.30	0.27	0.36	0.37	0.33	0.29	0.30	0.26	0.25	0.30
	**R2**	0.45	0.42	0.40	0.36	0.32	0.31	0.31	0.31	0.41	0.41	0.38	0.34	0.30	0.31	0.27	0.27	0.43	0.38	0.34	0.29	0.34	0.32	0.22	0.28
	**R3**	0.45	0.38	0.39	0.36	0.31	0.32	0.30	0.29	0.42	0.35	0.35	0.36	0.31	0.32	0.35	0.31	0.45	0.42	0.38	0.35	0.34	0.27	0.26	0.24
**Teicoplanin**	**R1**	0.00	0.00	0.00	0.00	0.00	0.00	0.00	0.00	0.00	0.00	0.00	0.00	0.00	0.00	0.00	0.00	0.00	0.00	0.00	0.00	0.00	0.00	0.00	0.00
	**R2**	0.00	0.00	0.00	0.00	0.00	0.00	0.00	0.00	0.00	0.00	0.00	0.00	0.00	0.00	0.00	0.00	0.00	0.00	0.00	0.00	0.00	0.00	0.00	0.00
	**R3**	0.00	0.00	0.00	0.00	0.00	0.00	0.00	0.00	0.00	0.00	0.00	0.00	0.00	0.00	0.00	0.00	0.00	0.00	0.00	0.00	0.00	0.00	0.00	0.00
**Growth control 2**	**C**	0.22±0.04								0.23±0.05								0.25±0.02							

Table S5. Absorbance values for *Escherichia coli* K12 C600 measured in the MHB medium with the addition of a given human lactoferrin (hLF)-derived peptide or colistin control antibiotic. Numbers from 128 to 1 represent peptide dilutions in µg/mL. Each colony has its own growth control. Growth control 1 corresponds to hLF 1-11 while Growth control 2 to hLF 397-412, hLF 448-464, hLF 668-683 and colistin. R1; R2; R3 – indicate repetitions. C – relates to the mean growth control for each colony.

E. coli		**Colony 1**								**Colony 2**								**Colony 3**							
		**128**	**64**	**32**	**16**	**8**	**4**	**2**	**1**	**128**	**64**	**32**	**16**	**8**	**4**	**2**	**1**	**128**	**64**	**32**	**16**	**8**	**4**	**2**	**1**
**hLF 1-11**	**R1**	0.37	0.38	0.51	0.56	0.51	0.48	0.57	0.52	0.33	0.40	0.56	0.57	0.56	0.58	0.56	0.56	0.40	0.31	0.48	0.49	0.46	0.44	0.49	0.48
	**R2**	0.35	0.44	0.51	0.48	0.48	0.49	0.49	0.49	0.34	0.45	0.54	0.56	0.55	0.70	0.58	0.57	0.36	0.46	0.50	0.50	0.51	0.45	0.46	0.46
	**R3**	0.36	0.38	0.52	0.52	0.56	0.50	0.57	0.49	0.29	0.40	0.53	0.56	0.54	0.56	0.60	0.56	0.35	0.37	0.53	0.48	0.49	0.50	0.47	0.63
**Growth control 1**	**C**	0.67±0.03								0.58±0.02								0.61±0.03							
**hLF 397-412**	**R1**	0.90	1.23	1.34	1.24	1.22	1.22	1.16	1.12	1.02	0.99	1.04	0.94	0.94	0.85	1.06	0.82	0.86	0.79	0.71	0.69	0.66	0.72	0.64	0.75
	**R2**	1.34	1.24	1.35	1.20	1.18	1.18	1.10	1.10	1.03	0.99	1.01	0.98	0.95	0.86	0.94	0.84	0.71	0.73	0.62	0.64	0.63	0.61	0.65	0.62
	**R3**	1.19	1.06	1.23	1.06	1.06	1.06	1.03	1.00	0.84	0.90	0.94	0.87	0.88	0.84	0.90	0.75	0.78	0.75	0.80	0.63	0.63	0.68	0.65	0.69
**hLF 448-464**	**R1**	0.65	0.66	0.72	0.64	0.65	0.64	0.62	0.59	0.57	0.60	0.60	0.57	0.57	0.55	0.55	0.55	0.57	0.56	0.58	0.66	0.64	0.66	0.67	0.67
	**R2**	0.66	0.65	0.66	0.67	0.64	0.63	0.64	0.63	0.59	0.58	0.59	0.59	0.59	0.59	0.65	0.55	0.55	0.62	0.58	0.57	0.63	0.66	0.72	0.68
	**R3**	0.74	0.72	0.77	0.70	0.71	0.66	0.62	0.75	0.63	0.64	0.62	0.62	0.58	0.57	0.69	0.59	0.70	0.68	0.66	0.66	0.67	0.66	0.69	0.70
**hLF 668-683**	**R1**	1.21	1.12	1.12	1.08	0.97	1.05	1.02	1.00	0.89	0.85	0.92	0.87	0.84	0.79	1.02	0.82	0.76	0.83	0.81	0.60	0.61	0.62	0.67	0.65
	**R2**	0.67	0.66	0.70	0.66	0.65	0.65	0.64	0.61	0.57	0.57	0.59	0.55	0.55	0.59	0.67	0.57	0.59	0.59	0.62	0.63	0.61	0.68	0.65	0.69
	**R3**	0.68	0.68	0.68	0.64	0.65	0.65	0.64	0.63	0.57	0.55	0.58	0.56	0.56	0.56	0.67	0.57	0.62	0.64	0.69	0.59	0.61	0.63	0.64	0.65
**Colistin**	**R1**	0.00	0.00	0.00	0.00	0.00	0.24	0.29	0.69	0.00	0.00	0.00	0.00	0.00	0.00	0.02	0.39	0.00	0.00	0.00	0.00	0.00	0.00	0.04	0.23
	**R2**	0.00	0.00	0.00	0.00	0.00	0.01	0.33	0.72	0.00	0.00	0.00	0.00	0.00	0.00	0.18	0.29	0.00	0.00	0.00	0.00	0.00	0.01	0.26	0.54
	**R3**	0.00	0.00	0.00	0.00	0.00	0.01	0.46	0.50	0.00	0.00	0.00	0.00	0.00	0.00	0.01	0.34	0.00	0.00	0.00	0.00	0.00	0.00	0.13	0.26
**Growth control 2**	**C**	0.67±0.03								0.58±0.02								0.61±0.03							

Table S6. Absorbance values for *Acinetobacter baumannii* ATCC 19606 measured in the MHB medium with the addition of a given human lactoferrin (hLF)-derived peptide or colistin control antibiotic. Numbers from 128 to 1 represent peptide dilutions in μg/mL. Each colony has its own growth control. Growth control 1 corresponds to hLF 1-11 while Growth control 2 to hLF 397-412, hLF 448-464, hLF 668-683 and colistin. R1; R2; R3 – indicate repetitions. C – relates to the mean growth control for each colony.

A. baumanii		**Colony 1**								**Colony 2**								**Colony 3**							
		**128**	**64**	**32**	**16**	**8**	**4**	**2**	**1**	**128**	**64**	**32**	**16**	**8**	**4**	**2**	**1**	**128**	**64**	**32**	**16**	**8**	**4**	**2**	**1**
**hLF 1-11**	**R1**	1.47	1.37	1.31	1.27	1.25	1.20	1.17	1.12	1.39	1.32	1.27	1.30	1.23	1.27	1.25	1.24	1.22	1.24	1.21	1.25	1.20	1.18	0.60	0.61
	**R2**	1.45	1.41	1.35	1.34	1.31	1.31	1.23	1.20	1.26	1.16	1.14	1.11	1.07	1.09	1.12	1.03	0.93	0.82	0.90	0.76	0.75	0.69	0.65	0.59
	**R3**	1.28	1.30	1.32	1.31	1.20	1.29	1.18	1.16	1.22	1.15	1.18	1.12	1.10	1.08	0.98	1.00	0.93	0.83	0.80	0.75	0.73	0.72	0.68	0.58
**Growth control 1**	**C**	0.82±0.01								0.87±0.02								0.96±0.07							
**hLF 397-412**	**R1**	1.35	1.42	1.35	1.26	1.23	1.25	1.23	1.24	1.19	1.16	1.16	1.23	1.07	1.05	1.14	1.14	0.97	0.83	0.84	0.77	0.60	0.60	0.64	1.19
	**R2**	1.40	1.30	1.26	1.24	1.34	1.47	1.23	1.22	1.20	1.16	1.16	1.09	1.10	1.05	1.03	1.03	0.90	0.80	0.78	0.73	0.59	0.59	0.64	1.16
	**R3**	1.29	1.28	1.20	1.15	1.14	1.12	1.10	1.10	1.08	1.08	1.06	1.09	0.99	0.95	1.03	0.99	0.87	0.74	0.75	0.63	0.78	0.78	0.88	0.93
**hLF 448-464**	**R1**	1.39	1.27	1.33	1.20	1.16	1.14	1.03	1.04	1.07	1.00	0.91	0.93	0.79	0.73	0.77	0.77	0.68	0.66	0.62	0.86	0.87	0.92	0.96	0.99
	**R2**	1.44	1.33	1.32	1.23	1.25	1.34	1.09	1.03	0.99	0.98	0.93	0.85	0.75	0.72	0.66	0.65	0.65	0.75	0.65	0.85	0.92	0.95	1.02	1.05
	**R3**	1.39	1.37	1.34	1.32	1.36	1.31	1.30	1.28	1.38	1.38	1.28	0.72	1.20	1.10	1.22	1.22	0.94	1.06	0.64	0.96	1.11	1.01	1.13	1.07
**hLF 668-683**	**R1**	1.33	1.23	1.22	1.18	1.21	1.32	1.01	1.07	1.13	1.08	1.04	0.99	0.99	0.97	0.92	0.90	0.75	0.83	0.74	0.79	0.82	0.82	0.84	0.94
	**R2**	1.32	1.24	1.22	1.21	1.16	1.19	1.16	0.94	0.95	0.93	0.86	0.95	0.80	0.80	0.84	0.83	0.65	0.68	0.70	0.63	0.80	0.83	0.90	0.91
	**R3**	1.44	1.29	1.24	1.23	1.27	1.40	1.20	1.04	0.94	0.93	0.88	0.82	0.78	0.76	0.71	0.72	0.68	0.66	0.62	0.63	0.84	0.87	0.94	0.98
**Colistin**	**R1**	0.00	0.00	0.00	0.00	0.00	0.03	0.01	1.22	0.00	0.00	0.00	0.00	0.00	0.00	0.00	0.00	0.00	0.00	0.00	0.00	0.00	0.00	0.00	0.05
	**R2**	0.00	0.00	0.00	0.00	0.00	0.00	0.00	1.11	0.00	0.00	0.00	0.00	0.00	0.00	0.00	1.35	0.00	0.00	0.00	0.00	0.00	0.00	0.47	0.72
	**R3**	0.00	0.00	0.00	0.00	0.00	0.01	0.05	1.17	0.00	0.00	0.00	0.00	0.00	0.00	0.00	0.00	0.00	0.00	0.00	0.00	0.00	0.00	0.02	0.52
**Growth control 2**	**C**	1.37±0.03								1.41±0.02								1.29±0.04							

Table S7. Absorbance values for *Pseudomonas aeruginosa* ATCC 27853 measured in the MHB medium with the addition of a given human lactoferrin (hLF)-derived peptide or colistin control antibiotic. Numbers from 128 to 1 represent peptide dilutions in µg/mL. Each colony has its own growth control. Growth control 1 corresponds to hLF 1-11 while Growth control 2 to hLF 397-412, hLF 448-464, hLF 668-683 and colistin. R1; R2; R3 – indicate repetitions. C – relates to the mean growth control for each colony.

P. aeruginosa		Colony 1								Colony 2								Colony 3							
		**128**	**64**	**32**	**16**	**8**	**4**	**2**	**1**	**128**	**64**	**32**	**16**	**8**	**4**	**2**	**1**	**128**	**64**	**32**	**16**	**8**	**4**	**2**	**1**
**hLF 1-11**	**R1**	0.00	0.14	1.31	1.13	1.05	1.08	1.12	1.13	0.08	0.23	1.09	1.33	1.26	1.35	1.36	1.30	0.21	0.19	0.82	1.15	1.17	1.12	1.11	0.97
	**R2**	0.00	0.22	1.30	1.09	1.11	1.02	1.09	1.10	0.10	0.05	1.19	1.37	1.33	1.37	1.35	1.34	0.03	0.20	0.67	0.96	0.96	0.90	0.87	0.83
	**R3**	0.02	0.12	1.32	1.21	1.16	1.17	1.06	1.11	0.00	0.15	1.25	1.34	1.36	1.35	1.34	1.27	0.01	0.02	0.44	0.87	0.93	0.88	0.86	0.81
**Growth control 1**	**C**	0.89±0.05								0.78±0.05								0.78±0.03							
**hLF 397-412**	**R1**	1.10	0.67	0.64	0.62	0.59	0.59	0.59	0.74	0.57	0.56	0.57	0.61	0.62	0.55	0.58	0.54	0.57	0.54	0.60	0.57	0.59	0.59	0.59	0.67
	**R2**	0.80	0.63	0.61	0.64	0.64	0.59	0.60	0.61	0.58	0.67	0.59	0.57	0.58	0.58	0.58	0.57	0.57	0.56	0.57	0.57	0.57	0.59	0.59	0.66
	**R3**	0.68	0.62	0.63	0.60	0.60	0.61	0.59	0.59	0.59	0.54	0.70	0.62	0.60	0.59	0.57	0.56	0.58	0.55	0.54	0.56	0.60	0.56	0.59	0.64
**hLF 448-464**	**R1**	0.69	0.65	0.63	0.62	0.63	0.63	0.62	0.60	0.57	0.62	0.60	0.62	0.61	0.61	0.60	0.60	0.59	0.60	0.60	0.61	0.60	0.60	0.59	0.63
	**R2**	0.67	0.60	0.61	0.63	0.61	0.55	0.71	0.55	0.63	0.55	0.61	0.62	0.58	0.61	0.60	0.62	0.60	0.59	0.59	0.60	0.61	0.59	0.61	0.65
	**R3**	0.87	0.75	0.74	0.73	0.72	0.71	0.73	0.71	0.69	0.72	0.71	0.71	0.68	0.64	0.70	0.69	0.71	0.68	0.70	0.71	0.60	0.59	0.59	0.65
**hLF 668-683**	**R1**	0.64	0.60	0.61	0.61	0.64	0.55	0.56	0.72	0.61	0.62	0.60	0.57	0.57	0.58	0.57	0.56	0.55	0.55	0.54	0.54	0.60	0.61	0.59	0.63
	**R2**	0.63	0.58	0.60	0.58	0.58	0.61	0.59	0.54	0.59	0.58	0.59	0.59	0.60	0.56	0.59	0.57	0.57	0.58	0.54	0.55	0.58	0.57	0.56	0.61
	**R3**	0.66	0.62	0.58	0.62	0.57	0.53	0.53	0.58	0.60	0.59	0.59	0.57	0.56	0.57	0.57	0.59	0.56	0.57	0.55	0.56	0.58	0.60	0.59	0.62
**Colistin**	**R1**	0.00	0.00	0.00	0.00	0.00	0.01	0.82	1.30	0.00	0.00	0.00	0.00	0.01	0.02	1.03	0.85	0.00	0.00	0.00	0.00	0.00	0.05	0.48	0.94
	**R2**	0.00	0.00	0.00	0.00	0.00	0.01	1.18	1.15	0.00	0.00	0.00	0.00	0.00	0.00	0.76	1.18	0.00	0.00	0.00	0.00	0.01	0.00	0.23	0.83
	**R3**	0.00	0.00	0.00	0.00	0.00	0.00	0.67	1.26	0.00	0.00	0.00	0.00	0.00	0.00	0.96	1.16	0.00	0.00	0.00	0.00	0.00	0.01	0.50	0.74
**Growth control 2**	**C**	0.71±0.04								0.71±0.03								0.77±0.07							

Table S8. Absorbance values for *Escherichia coli* K12 C600 measured in the MHB medium with the addition of a given human lactoferrin (hLF)-derived peptide or colistin control antibiotic with combination of cOmplete™ EDTA-free Protease Inhibitor Cocktail at 1X concentration. Numbers from 128 to 1 represent peptide dilutions in µg/mL and number 0 the mean control containg only bacteria with protease inhibitor cocktail. R1; R2; R3 – indicate repetitions. C – relates to the mean growth control for each colony.

E. coli		Colony 1									Colony 2								
		**128**	**64**	**32**	**16**	**8**	**4**	**2**	**1**	**0**	**128**	**64**	**32**	**16**	**8**	**4**	**2**	**1**	**0**
**hLF 1-11**	**R1**	0.52	0.50	0.43	0.44	0.39	0.42	0.40	0.42		0.49	0.43	0.39	0.38	0.40	0.53	0.50	0.54	
	**R2**	0.53	0.45	0.46	0.39	0.36	0.43	0.41	0.41		0.55	0.53	0.46	0.42	0.47	0.45	0.46	0.59	
	**R3**	0.50	0.40	0.42	0.42	0.41	0.41	0.41	0.40		0.51	0.51	0.54	0.44	0.45	0.51	0.45	0.55	
**hLF 397-412**	**R1**	0.32	0.40	0.38	0.43	0.42	0.40	0.40	0.40		0.41	0.39	0.38	0.39	0.42	0.38	0.40	0.40	
	**R2**	0.40	0.39	0.45	0.44	0.37	0.40	0.40	0.41		0.39	0.41	0.38	0.41	0.41	0.41	0.40	0.40	
	**R3**	0.40	0.43	0.39	0.42	0.40	0.40	0.39	0.40		0.37	0.41	0.34	0.40	0.40	0.39	0.41	0.40	
**hLF 448-464**	**R1**	0.44	0.40	0.39	0.39	0.43	0.39	0.41	0.41		0.42	0.43	0.42	0.43	0.43	0.41	0.41	0.41	
	**R2**	0.46	0.44	0.42	0.43	0.42	0.41	0.43	0.42	0.53±0.03	0.42	0.42	0.42	0.40	0.42	0.39	0.40	0.41	0.51±0.01
	**R3**	0.43	0.44	0.42	0.43	0.43	0.42	0.42	0.42		0.43	0.42	0.42	0.41	0.41	0.41	0.40	0.41	
**hLF 668-683**	**R1**	0.44	0.45	0.44	0.42	0.42	0.42	0.42	0.42		0.41	0.41	0.40	0.42	0.42	0.39	0.41	0.41	
	**R2**	0.44	0.45	0.46	0.42	0.44	0.43	0.43	0.43		0.45	0.43	0.43	0.42	0.42	0.43	0.41	0.43	
	**R3**	0.52	0.51	0.48	0.47	0.47	0.46	0.46	0.48		0.47	0.49	0.47	0.47	0.47	0.45	0.47	0.46	
**Colistin**	**R1**	0.00	0.00	0.00	0.00	0.00	0.00	0.01	0.74		0.00	0.00	0.00	0.00	0.00	0.00	0.01	0.69	
	**R2**	0.00	0.00	0.00	0.00	0.00	0.00	0.01	0.63		0.00	0.00	0.00	0.00	0.00	0.01	0.02	0.67	
	**R3**	0.00	0.00	0.00	0.01	0.00	0.00	0.01	0.75		0.00	0.00	0.00	0.00	0.00	0.00	0.03	0.70	
**Growth control**	**C**	0.61±0.06									0.60±0.03								

Table S9. Absorbance values for *Pseudomonas aeruginosa* ATCC 27853 measured in the MHB medium with the addition of a given human lactoferrin (hLF)-derived peptide or colistin control antibiotic with combination of cOmplete™ EDTA-free Protease Inhibitor Cocktail at 1X concentration. Numbers from 128 to 1 represent peptide dilutions in µg/mL and number 0 the mean control containg only bacteria with protease inhibitor cocktail R1; R2; R3 – indicate repetitions. C – relates to the mean growth control for each colony.

P. aeruginosa		Colony 1									Colony 2								
		**128**	**64**	**32**	**16**	**8**	**4**	**2**	**1**	**0**	**128**	**64**	**32**	**16**	**8**	**4**	**2**	**1**	**0**
**hLF 1-11**	**R1**	0.01	0.09	0.62	0.82	0.64	0.68	0.72	0.75	0.63±	0.00	0.04	0.33	0.54	0.44	0.53	0.55	0.57	0.59±

	**R2**	0.02	0.11	0.61	0.79	0.65	0.67	0.68	0.72	0.03	0.00	0.05	0.30	0.53	0.51	0.55	0.55	0.56	0.01
	**R3**	0.00	0.04	0.24	0.43	0.51	0.54	0.57	0.57		0.00	0.03	0.21	0.39	0.48	0.52	0.53	0.46	
**hLF 397-412**	**R1**	0.60	0.51	0.47	0.47	0.46	0.49	0.49	0.58		0.66	0.60	0.59	0.56	0.55	0.54	0.53	0.49	
	**R2**	0.59	0.53	0.50	0.49	0.48	0.46	0.44	0.56		0.59	0.52	0.47	0.47	0.45	0.46	0.46	0.47	
	**R3**	0.57	0.49	0.48	0.47	0.46	0.47	0.47	0.57		0.61	0.51	0.48	0.46	0.46	0.46	0.45	0.44	
**hLF 448-464**	**R1**	0.61	0.53	0.49	0.47	0.46	0.45	0.45	0.44		0.60	0.50	0.48	0.45	0.44	0.44	0.42	0.44	
	**R2**	0.60	0.53	0.49	0.48	0.51	0.46	0.43	0.46		0.59	0.50	0.49	0.47	0.47	0.47	0.45	0.43	
	**R3**	0.62	0.54	0.50	0.48	0.47	0.46	0.46	0.45		0.59	0.53	0.50	0.48	0.46	0.46	0.45	0.45	
**hLF 668-683**	**R1**	0.59	0.53	0.48	0.47	0.48	0.47	0.47	0.46		0.58	0.51	0.48	0.47	0.46	0.46	0.47	0.47	
	**R2**	0.58	0.55	0.54	0.50	0.49	0.49	0.47	0.48		0.61	0.52	0.50	0.50	0.48	0.48	0.49	0.51	
	**R3**	0.60	0.54	0.55	0.52	0.52	0.52	0.51	0.52		0.72	0.56	0.53	0.52	0.51	0.51	0.51	0.52	
**Colistin**	**R1**	0.00	0.00	0.00	0.00	0.00	0.00	0.00	1.01		0.00	0.00	0.00	0.00	0.00	0.00	0.03	1.09	
	**R2**	0.00	0.00	0.00	0.00	0.00	0.00	0.01	0.69		0.00	0.00	0.00	0.00	0.00	0.00	0.01	0.70	
	**R3**	0.00	0.00	0.00	0.00	0.00	0.00	0.01	0.75		0.00	0.00	0.00	0.00	0.00	0.00	0.03	0.83	
**Growth control**	**C**	0.83±0.05									0.84±0.03								

Table S10. Absorbance values for *Acinetobacter baumannii* ATCC 19606 measured in the MHB medium with the addition of a given human lactoferrin (hLF)-derived peptide or colistin control antibiotic with combination of cOmplete™ EDTA-free Protease Inhibitor Cocktail at 1X concentration. Numbers from 128 to 1 represent peptide dilutions in μg/mL and number 0 the mean control containg only bacteria with protease inhibitor cocktail. R1; R2; R3 – indicate repetitions. C – relates to the mean growth control for each colony.

A. baumanii		**Colony 1**									**Colony 2**								
		**128**	**64**	**32**	**16**	**8**	**4**	**2**	**1**	**0**	**128**	**64**	**32**	**16**	**8**	**4**	**2**	**1**	**0**
**hLF 1-11**	**R1**	0.66	0.60	0.62	0.59	0.55	0.53	0.59	0.54	0.54±0.02	0.50	0.52	0.53	0.51	0.49	0.52	0.52	0.54	0.53±0.04
	**R2**	0.72	0.61	0.59	0.55	0.55	0.51	0.51	0.52		0.51	0.55	0.52	0.51	0.52	0.50	0.50	0.55	
	**R3**	0.56	0.55	0.52	0.52	0.50	0.50	0.51	0.51		0.51	0.50	0.53	0.52	0.54	0.51	0.51	0.52	
**hLF 397-412**	**R1**	0.64	0.52	0.55	0.52	0.51	0.53	0.53	0.52		0.46	0.50	0.50	0.49	0.50	0.49	0.49	0.49	
	**R2**	0.55	0.53	0.52	0.53	0.53	0.51	0.53	0.52		0.51	0.48	0.51	0.52	0.51	0.52	0.51	0.54	

	**R3**	0.64	0.53	0.55	0.51	0.51	0.49	0.52	0.50		0.49	0.52	0.50	0.50	0.51	0.49	0.53	0.52	
**hLF 448-464**	**R1**	0.57	0.50	0.52	0.52	0.51	0.51	0.54	0.52		0.51	0.50	0.50	0.51	0.50	0.54	0.50	0.54	
	**R2**	0.64	0.52	0.54	0.52	0.51	0.51	0.55	0.53		0.53	0.53	0.53	0.51	0.52	0.52	0.51	0.52	
	**R3**	0.58	0.57	0.57	0.55	0.54	0.54	0.56	0.56		0.53	0.53	0.52	0.56	0.53	0.57	0.59	0.65	
**hLF 668-683**	**R1**	0.64	0.52	0.55	0.53	0.52	0.51	0.53	0.53		0.54	0.55	0.59	0.53	0.60	0.58	0.60	0.63	
	**R2**	0.62	0.62	0.65	0.64	0.65	0.63	0.63	0.64		0.50	0.50	0.52	0.53	0.52	0.50	0.52	0.53	
	**R3**	0.69	0.65	0.66	0.66	0.67	0.64	0.64	0.65		0.53	0.54	0.57	0.56	0.58	0.58	0.58	0.59	
**Colistin**	**R1**	0.00	0.00	0.00	0.00	0.00	0.00	0.09	0.91		0.00	0.00	0.00	0.00	0.00	0.00	0.08	0.64	
	**R2**	0.00	0.00	0.00	0.00	0.00	0.00	0.09	0.64		0.00	0.00	0.00	0.00	0.00	0.00	0.08	0.66	
	**R3**	0.00	0.00	0.00	0.00	0.00	0.00	0.03	0.74		0.00	0.00	0.00	0.00	0.00	0.00	0.06	0.64	
**Growth control**	**C**	1.16±0.10									1.09±0.04								

Table S11. Absorbance values for *Staphylococcus aureus* ATCC 25923 measured in the MHB medium with the addition of given concentrations of human lactoferrin (hLF)-derived or teicoplanin control antibiotic with combination of cOmplete™ EDTA-free Protease Inhibitor Cocktail at 1X concentration. Numbers from 128 to 1 represent peptide dilutions in µg/mL and number 0 the mean control containg only bacteria with protease inhibitor cocktail. R1; R2; R3 – indicate repetitions. C – relates to the mean growth control for each colony.

S. Aureus		**Colony 1**									**Colony 2**								
		**128**	**64**	**32**	**16**	**8**	**4**	**2**	**1**	**0**	**128**	**64**	**32**	**16**	**8**	**4**	**2**	**1**	**0**
**hLF 1-11**	**R1**	0.39	0.42	0.42	0.42	0.46	0.44	0.47	0.47	0.45± 0.03	0.42	0.43	0.44	0.43	0.44	0.45	0.44	0.45	0.47± 0.02
	**R2**	0.39	0.40	0.41	0.41	0.42	0.42	0.45	0.46		0.41	0.42	0.42	0.44	0.41	0.44	0.45	0.46	
	**R3**	0.42	0.42	0.39	0.43	0.42	0.47	0.37	0.47		0.43	0.42	0.45	0.44	0.45	0.45	0.45	0.46	
**hLF 397-412**	**R1**	0.42	0.42	0.42	0.40	0.40	0.41	0.40	0.49		0.40	0.41	0.40	0.42	0.40	0.41	0.42	0.42	
	**R2**	0.41	0.41	0.46	0.40	0.42	0.39	0.42	0.46		0.43	0.40	0.40	0.40	0.43	0.39	0.40	0.43	
	**R3**	0.42	0.40	0.40	0.44	0.42	0.42	0.42	0.46		0.42	0.44	0.40	0.40	0.40	0.40	0.41	0.40	
**hLF 448-464**	**R1**	0.44	0.45	0.45	0.46	0.54	0.53	0.52	0.52		0.44	0.42	0.46	0.46	0.47	0.48	0.53	0.54	
	**R2**	0.43	0.47	0.49	0.49	0.54	0.50	0.50	0.48		0.46	0.47	0.48	0.46	0.51	0.50	0.52	0.54	
	**R3**	0.44	0.46	0.48	0.50	0.55	0.52	0.54	0.53		0.46	0.48	0.50	0.47	0.50	0.51	0.53	0.54	

**hLF 668-683**	**R1**	0.49	0.46	0.42	0.44	0.47	0.45	0.47	0.47		0.47	0.46	0.45	0.43	0.43	0.47	0.46	0.45	
	**R2**	0.47	0.47	0.46	0.45	0.44	0.45	0.45	0.47		0.51	0.49	0.47	0.46	0.47	0.48	0.47	0.47	
	**R3**	0.50	0.49	0.49	0.47	0.49	0.45	0.42	0.46		0.51	0.50	0.48	0.50	0.47	0.47	0.48	0.48	
**Teicoplanin**	**R1**	0.03	0.00	0.01	0.00	0.00	0.00	0.00	0.00		0.00	0.00	0.00	0.00	0.00	0.00	0.00	0.00	
	**R2**	0.00	0.00	0.00	0.00	0.00	0.00	0.00	0.00		0.00	0.00	0.00	0.00	0.00	0.00	0.00	0.00	
	**R3**	0.00	0.00	0.00	0.00	0.00	0.00	0.00	0.00		0.00	0.00	0.00	0.00	0.00	0.00	0.00	0.00	
**Growth control**	**C**	0.60±0.02									0.62±0.01								

Table S12. Absorbance values for *Enterococcus faecium* PCM1859 measured in the MHB medium with the addition of given concentrations of human lactoferrin (hLF)-derived or teicoplanin control antibiotic with combination of cOmplete™ EDTA-free Protease Inhibitor Cocktail at 1X concentration. Numbers from 128 to 1 represent peptide dilutions in μg/mL and number 0 the mean control containg only bacteria with protease inhibitor cocktail. R1; R2; R3 – indicate repetitions. C – relates to the mean growth control for each colony.

E. faecium		Colony 1									Colony 2								
		**128**	**64**	**32**	**16**	**8**	**4**	**2**	**1**	**0**	**128**	**64**	**32**	**16**	**8**	**4**	**2**	**1**	**0**
**hLF 1-11**	**R1**	0.52	0.47	0.56	0.45	0.44	0.47	0.46	0.43	0.41±<br>0.01	0.38	0.40	0.43	0.43	0.41	0.43	0.42	0.43	0.42±<br>0.01
	**R2**	0.52	0.50	0.49	0.46	0.45	0.46	0.45	0.44		0.40	0.42	0.42	0.43	0.43	0.44	0.45	0.43	
	**R3**	0.53	0.50	0.46	0.46	0.45	0.44	0.44	0.44		0.42	0.42	0.44	0.43	0.45	0.44	0.45	0.45	
**hLF 397-412**	**R1**	0.44	0.46	0.46	0.45	0.44	0.42	0.38	0.40		0.45	0.45	0.46	0.45	0.47	0.46	0.45	0.46	
	**R2**	0.47	0.47	0.49	0.47	0.46	0.42	0.39	0.42		0.46	0.47	0.47	0.46	0.45	0.47	0.46	0.48	
	**R3**	0.47	0.48	0.49	0.47	0.44	0.42	0.39	0.40		0.45	0.48	0.48	0.47	0.47	0.46	0.46	0.47	
**hLF 448-464**	**R1**	0.52	0.52	0.50	0.50	0.48	0.45	0.38	0.45		0.50	0.48	0.49	0.48	0.48	0.48	0.48	0.49	
	**R2**	0.50	0.51	0.50	0.51	0.44	0.43	0.38	0.44		0.51	0.48	0.49	0.48	0.47	0.48	0.47	0.49	
	**R3**	0.54	0.51	0.51	0.51	0.48	0.47	0.42	0.47		0.51	0.49	0.49	0.49	0.47	0.48	0.49	0.51	
**hLF 668-683**	**R1**	0.47	0.45	0.45	0.46	0.44	0.42	0.42	0.43		0.47	0.45	0.43	0.44	0.44	0.44	0.44	0.45	
	**R2**	0.51	0.45	0.46	0.46	0.47	0.48	0.43	0.50		0.43	0.42	0.43	0.41	0.44	0.43	0.44	0.44	
	**R3**	0.39	0.40	0.41	0.40	0.43	0.44	0.44	0.48		0.42	0.41	0.40	0.40	0.41	0.40	0.39	0.39	
**Teicoplanin**	**R1**	0.00	0.00	0.00	0.00	0.00	0.00	0.00	0.00		0.00	0.00	0.00	0.00	0.00	0.00	0.00	0.00	

	R2	0.00	0.00	0.00	0.00	0.00	0.00	0.00	0.00		0.00	0.00	0.00	0.00	0.00	0.00	0.00	0.00	
	R3	0.00	0.00	0.00	0.00	0.00	0.00	0.00	0.00		0.00	0.00	0.00	0.00	0.00	0.00	0.00	0.00	
**Growth control**	**C**	0.44±0.02									0.43±0.01								

Table S13. Absorbance values for *Enterococcus faecalis* ATCC 29212 measured in the MHB medium with the addition of given concentrations of human lactoferrin (hLF)-derived or teicoplanin control antibiotic with combination of cOmplete™ EDTA-free Protease Inhibitor Cocktail at 1X concentration. Numbers from 128 to 1 represent peptide dilutions in µg/mL and number 0 the mean control containg only bacteria with protease inhibitor cocktail. R1; R2; R3 – indicate repetitions. C – relates to the mean growth control for each colony.

E. faecalis		**Colony 1**									**Colony 2**								
		**128**	**64**	**32**	**16**	**8**	**4**	**2**	**1**	**0**	**128**	**64**	**32**	**16**	**8**	**4**	**2**	**1**	**0**
**hLF 1-11**	**R1**	0.41	0.37	0.38	0.29	0.24	0.23	0.23	0.23		0.29	0.31	0.31	0.27	0.24	0.26	0.22	0.21	
	**R2**	0.34	0.38	0.33	0.28	0.24	0.25	0.23	0.23		0.29	0.34	0.30	0.26	0.23	0.23	0.24	0.22	
	**R3**	0.35	0.37	0.33	0.26	0.23	0.22	0.21	0.21		0.30	0.35	0.32	0.28	0.22	0.22	0.22	0.20	
**hLF 397-412**	**R1**	0.21	0.20	0.21	0.22	0.22	0.22	0.22	0.21		0.21	0.21	0.20	0.21	0.21	0.21	0.20	0.20	
	**R2**	0.22	0.21	0.22	0.22	0.21	0.22	0.21	0.22		0.23	0.21	0.21	0.21	0.21	0.21	0.20	0.21	
	**R3**	0.21	0.22	0.21	0.22	0.22	0.21	0.21	0.21		0.21	0.21	0.20	0.20	0.21	0.21	0.21	0.20	
**hLF 448-464**	**R1**	0.23	0.25	0.24	0.23	0.21	0.21	0.20	0.21		0.24	0.23	0.22	0.21	0.21	0.22	0.21	0.21	
	**R2**	0.22	0.24	0.24	0.23	0.22	0.22	0.22	0.22	0.24±0.01	0.25	0.23	0.22	0.24	0.23	0.21	0.21	0.20	0.23±0.01
	**R3**	0.25	0.25	0.25	0.24	0.23	0.22	0.21	0.21		0.25	0.23	0.23	0.22	0.22	0.22	0.21	0.21	
**hLF 668-683**	**R1**	0.22	0.22	0.23	0.23	0.23	0.23	0.22	0.22		0.23	0.21	0.21	0.25	0.25	0.22	0.21	0.21	
	**R2**	0.22	0.27	0.24	0.23	0.23	0.23	0.22	0.23		0.24	0.22	0.23	0.22	0.21	0.23	0.22	0.22	
	**R3**	0.25	0.22	0.23	0.23	0.23	0.23	0.23	0.23		0.24	0.22	0.22	0.27	0.25	0.22	0.22	0.22	
**Teicoplanin**	**R1**	0.00	0.00	0.00	0.00	0.00	0.00	0.00	0.00		0.00	0.00	0.00	0.00	0.00	0.00	0.01	0.00	
	**R2**	0.00	0.00	0.00	0.00	0.00	0.00	0.00	0.00		0.00	0.00	0.00	0.00	0.00	0.00	0.00	0.00	
	**R3**	0.00	0.00	0.00	0.00	0.00	0.00	0.00	0.00		0.00	0.00	0.00	0.00	0.00	0.00	0.00	0.00	
**Growth control**	**C**	0.29±0.01									0.28±0.01								

Table S14. Absorbance values for *Escherichia coli* K12 C600 measured in the MHB medium with the addition of a given human lactoferrin (hLF)-derived peptide or colistin control antibiotic with combination of 1 mM EDTA. Numbers from 128 to 1 represent peptide dilutions in μg/mL and number 0 the mean control containg only bacteria with EDTA. R1; R2; R3 – indicate repetitions. C – relates to the mean growth control.

E. coli		128	64	32	16	8	4	2	1	0
hLF 1-11	R1	0.00	0.00	0.00	0.19	0.13	0.25	0.08	0.12	0.25±0.04
	R2	0.00	0.00	0.00	0.00	0.21	0.21	0.12	0.13	
	R3	0.00	0.00	0.00	0.12	0.27	0.25	0.15	0.17	
hLF 397-412	R1	0.12	0.15	0.22	0.20	0.17	0.23	0.16	0.15	
	R2	0.13	0.20	0.21	0.16	0.19	0.18	0.23	0.20	
	R3	0.17	0.23	0.23	0.20	0.19	0.20	0.20	0.23	
hLF 448-464	R1	0.18	0.20	0.23	0.14	0.23	0.25	0.23	0.25	
	R2	0.16	0.19	0.20	0.21	0.21	0.26	0.24	0.19	
	R3	0.20	0.21	0.24	0.18	0.21	0.23	0.25	0.18	
hLF 668-683	R1	0.18	0.17	0.22	0.21	0.19	0.23	0.23	0.18	
	R2	0.17	0.20	0.19	0.21	0.20	0.20	0.25	0.22	
	R3	0.18	0.19	0.23	0.18	0.15	0.29	0.28	0.17	
Colistin	R1	0.00	0.00	0.00	0.00	0.00	0.00	0.29	0.18	
	R2	0.00	0.00	0.00	0.00	0.00	0.00	0.19	0.22	
	R3	0.00	0.00	0.00	0.00	0.00	0.00	0.23	0.17	
Growth control	C	1.25±0.08								

Table S15. Absorbance values for *Staphylococcus aureus* ATCC 25923 measured in the MHB medium with the addition of given concentrations of human lactoferrin (hLF)-derived or teicoplanin control antibiotic with combination of 1 mM EDTA. Numbers from 128 to 1 represent peptide dilutions in μg/mL and number 0 the mean control containg only bacteria with EDTA. R1; R2; R3 – indicate repetitions. C – relates to the mean growth control.

S. aureus		128	64	32	16	8	4	2	1	0
**hLF 1-11**	**R1**	0.04	0.13	0.13	0.14	0.14	0.20	0.26	0.26	0.23±0.01
	**R2**	0.05	0.13	0.13	0.12	0.13	0.15	0.21	0.26	
	**R3**	0.05	0.11	0.14	0.12	0.12	0.14	0.22	0.23	
**hLF 397-412**	**R1**	0.12	0.12	0.19	0.15	0.20	0.20	0.22	0.23	
	**R2**	0.12	0.13	0.13	0.21	0.21	0.21	0.23	0.24	
	**R3**	0.12	0.11	0.14	0.15	0.18	0.18	0.20	0.17	
**hLF 448-464**	**R1**	0.12	0.11	0.15	0.17	0.26	0.20	0.25	0.24	
	**R2**	0.13	0.15	0.19	0.19	0.22	0.21	0.25	0.23	
	**R3**	0.12	0.12	0.18	0.22	0.21	0.22	0.25	0.23	
**hLF 668-683**	**R1**	0.12	0.11	0.20	0.20	0.23	0.23	0.23	0.21	
	**R2**	0.13	0.13	0.16	0.22	0.22	0.23	0.25	0.23	
	**R3**	0.14	0.15	0.18	0.19	0.20	0.20	0.22	0.20	
**Teicoplanin**	**R1**	0.00	0.00	0.00	0.01	0.00	0.00	0.01	0.01	
	**R2**	0.00	0.01	0.00	0.01	0.00	0.00	0.01	0.01	
	**R3**	0.00	0.01	0.00	0.00	0.00	0.00	0.01	0.01	
**Growth control**	**C**	0.53±0.04								

Table S16. Absorbance values for *Staphylococcus aureus* ATCC 25923 measured in the MHB medium with the addition of given concentrations of human lactoferrin (hLF)-derived or teicoplanin control antibiotic with combination of cOmplete™ Protease Inhibitor Cocktail at 1X concentration (with EDTA). Numbers from 128 to 1 represent peptide dilutions in μg/mL and number 0 the mean control containg only bacteria with Protease Inhibitor Cocktail. R1; R2; R3 – indicate repetitions. C – relates to the mean growth control.

S. aureus		128	64	32	16	8	4	2	1	0
**hLF 1-11**	**R1**	0.03	0.02	0.02	0.02	0.01	0.01	0.02	0.02	0.03±0.01
	**R2**	0.03	0.02	0.02	0.01	0.01	0.01	0.03	0.01	
	**R3**	0.04	0.02	0.02	0.02	0.01	0.01	0.01	0.01	
**hLF 397-412**	**R1**	0.05	0.03	0.03	0.01	0.02	0.02	0.00	0.01	
	**R2**	0.05	0.04	0.03	0.02	0.02	0.02	0.01	0.01	
	**R3**	0.05	0.04	0.03	0.02	0.02	0.02	0.03	0.03	
**hLF 448-464**	**R1**	0.09	0.05	0.04	0.03	0.03	0.03	0.02	0.02	
	**R2**	0.14	0.05	0.03	0.02	0.02	0.02	0.02	0.02	
	**R3**	0.12	0.05	0.03	0.03	0.03	0.02	0.02	0.02	
**hLF 668-683**	**R1**	0.12	0.04	0.03	0.03	0.03	0.02	0.02	0.02	
	**R2**	0.06	0.04	0.05	0.07	0.03	0.02	0.02	0.02	
	**R3**	0.06	0.04	0.03	0.02	0.02	0.02	0.02	0.02	
**Teicoplanin**	**R1**	0.00	0.00	0.00	0.00	0.01	0.00	0.00	0.02	
	**R2**	0.00	0.00	0.00	0.00	0.00	0.00	0.00	0.00	
	**R3**	0.00	0.00	0.00	0.00	0.00	0.00	0.00	0.00	
**Growth control**	**C**	0.61±0.04								

Table S17. Experimentally confirmed non-AMPs.

UniProt entry	Protein name	Organism	Length
O93454	Plasticin-DA1	*Agalychnis dacnicolor*	71
Q8ISL8	Apisimin	*Apis mellifera*	78
E4Z7G0	Kassorin-M	*Phlyctimantis maculatus*	65
C0HJV7	Met-lysine-1a	*Lachesana tarabaevi*	121
C0HJV8	Met-lysine-1b	*Lachesana tarabaevi*	121
A0A1W6EVN2	Ampulexin 2	*Ampulex compressa*	50
A0A1W6EVM7	Ampulexin 1	*Ampulex compressa*	50
C0HLE0	Plasticin-TR	*Phyllomedusa trinitatis*	22
P85507	Ranaspumin	*Leptodactylus vastus*	217
P84270	Dahlein-5.4	*Ranoidea dahlii*	21
P84267	Dahlein-5.1	*Ranoidea dahlii*	20
P84272	Dahlein-5.6	*Ranoidea dahlii*	21
P84268	Dahlein-5.2	*Ranoidea dahlii*	21
P84269	Dahlein-5.3	*Ranoidea dahlii*	20
P84271	Dahlein-5.5	*Ranoidea dahlii*	21
B3KYH5	Temporin-SHb	*Pelophylax saharicus*	50
P0DTV3	Lesueurin	*Ranoidea lesueuri*	13
P86129	Riparin-5.1	*Crinia riparia*	16
C0HJK1	U1-poneritoxin-Dq1c	*Dinoponera quadriceps*	9
C0HK83	Magainin-B1	*Xenopus borealis*	21
P84265	Dahlein-4.2	*Ranoidea dahlii*	23
P84266	Dahlein-4.3	*Ranoidea dahlii*	23
P84264	Dahlein-4.1	*Ranoidea dahlii*	23
P69033	Splendipherin	*Ranoidea splendida*	25
P10636	Microtubule-associated protein tau-derived fragment(VQIVCK)	*Homo sapiens*	6
P10636	Microtubule-associated protein tau-derived fragment(VCIVYK)	*Homo sapiens*	6
P10636	Microtubule-associated protein tau-derived fragment (VKIVYK)	*Homo sapiens*	6
P10636	Microtubule-associated protein tau-derived fragment (VGIVYK)	*Homo sapiens*	6
P05067	Amyloid-beta precursor protein-derived fragment (GAIIGL)	*Homo sapiens*	6
P05067	Amyloid-beta precursor protein-derived fragment (AIIGLM)	*Homo sapiens*	6
P02511	Alpha-crystallin B chain-derived fragment (LKVKVL)	*Homo sapiens*	6
P04156	Major prion protein-derived fragment (GGYLLG)	*Homo sapiens*	6
n/d	n/d (LIVAGK)	*n/d*	6
n/d	n/d (KCWCFT)	*n/d*	6
